# Electrocardiographic criteria for localization of ventricular premature complexes from the inferior right ventricular outflow tract

**DOI:** 10.3389/fcvm.2022.950401

**Published:** 2022-10-10

**Authors:** Kang Li, Pinchao Lv, Yuchuan Wang, Fangfang Fan, Yansheng Ding, Jianping Li, Jing Zhou

**Affiliations:** Department of Cardiology, Peking University First Hospital, Beijing, China

**Keywords:** ventricular premature complexes, inferior right ventricular outflow tract, catheter ablation, electrocardiogram, algorithms

## Abstract

**Background:**

The ventricular premature complexes (PVCs) originating from the superior right ventricular outflow tract (RVOT) have high success rates by catheter ablation. It may not be the same when the origin is in the inferior RVOT.

**Objective:**

To identify electrocardiographic (ECG) characteristics that predict the site for successful ablation of PVCs originating in the inferior RVOT.

**Methods:**

Of 309 consecutive patients with symptomatic PVCs despite medical therapy, 124 had PVCs originating from the RVOT, and 107 RVOT cases without structural heart disease and no bundle branch block in sinus rhythm were enrolled in the study. Among them, 74 have a superior RVOT origin, and 33 have an inferior RVOT origin.

**Results:**

The proportion with multiple morphologies of PVC was significantly higher in the inferior RVOT group than in the superior RVOT group (24.24 vs. 6.76%, *P* = 0.011). The QRS duration of PVCs with an inferior RVOT origin was more expansive than PVCs with a superior RVOT origin (162.42 ± 19.69 ms vs. 140.90 ± 11.30 ms; *P* < 0.001). Furthermore, the QRS wave in V1 in patients in the inferior RVOT group was more likely to have a negative delta wave at the onset of the QRS (27.27 vs. 1.39%, *P* < 0.001). We found that the areas under the receiver-operating characteristic curve (AUCs) for PVC diagnosis with an inferior RVOT origin ranged from 0.812 to 0.841 depending on ECG features, with the highest AUC for the QRS duration of PVCs and the amplitude of R waves in lead II. These ECG indices had good predictability for judging the origin of PVCs in the RVOT; the best threshold for the QRS duration of PVCs was 145 ms, and the best thresholds for the amplitude of R waves in leads II, III, and aVF were 1.35, 1.35, and 1.15 mV, respectively.

**Conclusion:**

When evaluating a patient with PVCs, the source is likely to be the inferior RVOT if the ECG presentation conforms to the morphological characteristics of the RVOT, meanwhile, the QRS wave is relatively broad and polymorphic, and the main waves in limb leads (II, III, and aVF) are upward with low amplitude.

## Introduction

Ventricular premature complexes (PVCs) in patients with structurally normal hearts usually originate from the right ventricular outflow tract (RVOT). Catheter ablation is an excellent treatment option for these PVCs, with a success rate of >80% and few significant complications (1). The RVOT is bounded by the supraventricular crest and the pulmonic valve (2). Previous studies have demonstrated that the origin of PVCs from the RVOT is mostly above or below the pulmonary sinus cusp (3). Moreover, Zhang et al. reported that reverse U-curve ablation above the pulmonary sinus cusp could improve the treatment success rate (4). However, there are still some cases of failure or recurrence of PVCs from the RVOT after catheter ablation, especially when the origin is remote from the pulmonary annulus. In patients with failed ablation of idiopathic left bundle branch block (LBBB) and PVCs with an inferior axis morphology, the most frequent origin is still in the RVOT, followed by an intramural focus and the pulmonary artery (5, 6). In these patients, the septum of the inferior RVOT is a site where ablation occasionally fails. The septal papillary muscles, abundant trabeculae, and moderator band intersect at the junction of the smooth-walled pulmonary infundibulum and the large trabecular inflow tract, making it difficult for the catheter to attach (6).

The electrocardiogram (ECG) in patients with PVCs originating from the RVOT shows the characteristics of LBBB and an inferior axis morphology (7). However, the ECG characteristics of inferior RVOT have rarely been described. ECG-guided prediction of localization of RVOT has important procedural implications concerning the selection of catheter and auxiliary sheath, procedure time, and risk of complications. In this study, we sought to identify ECG characteristics that can predict the site for successful ablation of PVCs originating from the inferior RVOT.

## Methods

### Study population

This is a retrospective study. Between January 2016 and December 2020, 309 consecutive patients at Peking University First Hospital were identified to have symptomatic PVCs despite medical therapy. One hundred and twenty-four of these patients had PVCs originating in the RVOT. Among these RVOT cases, 107 patients without structural heart disease and no bundle branch block in sinus rhythm, were enrolled in the study. Left and right ventricular function was assessed by echocardiography before catheter ablation in all patients. From April 2019 onwards, we used intracardiac echocardiography (ICE) in PVC ablations.

### ECG evaluation

Twelve-lead ECGs of symptomatic PVCs were analyzed for bundle branch block morphology, axis, QRS width, notching in V1–V6, R-wave pattern in V1 and V2 (rS, QS), and the precordial transition point from predominantly negative S-wave to predominantly positive R-wave deflection. Early QRS transition was defined as a transition in V3 or earlier and late transition as a transition in V4 or V5. Notching was defined as deflections in the QRS complex aside from a triphasic pattern.

### Electrophysiological study

All patients underwent invasive electrophysiologic testing under local anesthesia in an awake state after providing written informed consent. Antiarrhythmic medications were discontinued at least five half-lives before the procedure, and beta-blockers and calcium channel blockers were withheld for at least 3 days. The right ventricle was mapped via the standard femoral approach. Intravenous isoproterenol (up to 10 μg/min for 5–10 min) was administered as necessary to induce ectopy.

### Definition of superior and inferior RVOT

We considered a superior RVOT origin to be within 1 cm above or below the pulmonary annulus. An inferior RVOT origin is between the supraventricular crest and the pulmonary infundibulum and more than 1 cm away from the pulmonary annulus (8).

### Mapping and ablation

Three-dimensional electroanatomic mapping (Carto; Biosense Webster Inc., Diamond Bar, CA, USA) was performed during all procedures. A 6-F steerable decapolar catheter (Dynamic XT; Boston Scientific, Marlborough, MA, USA) was advanced into the coronary sinus. An 8.5-F long sheath (St Jude Medical, St Paul, MN, USA) was used to facilitate and stabilize the mapping catheter. In 15 patients (7 patients in the superior RVOT group, and 8 patients in the inferior RVOT group), a 10-F ICE catheter (SoundStar; Biosense Webster Inc.) was advanced to the right ventricle to assist with mapping and ablation and monitor for complications. Mapping and radiofrequency ablation were performed through an open irrigated ablation catheter (Thermocool SmartTouch or Navistar; Biosense Webster Inc.). Anatomic and activation mapping was performed simultaneously using the three-dimensional Carto mapping system. The earliest point of activation during clinical PVCs was targeted for ablation. If PVC suppression was observed during ablation, additional radiofrequency energy was applied at or adjacent to the successful ablation site. Radiofrequency energy was applied for at least 45–60 sec per site using 30–35 W. The number of lesions delivered was in the range of 5–9. Patients were monitored for a minimum of 15–30 min after the last radiofrequency application to eliminate PVCs. Patients were monitored overnight on the day of the procedure and discharged on the following day. The procedural endpoint was the complete elimination of clinical PVCs following catheter ablation. Suppression of the PVC burden by >80% on 24-h Holter monitoring immediately after the procedure was defined as an immediate success.

### Follow-up

Patients were followed up for 3 months post-ablation. A 24-h Holter monitor was performed 1 and 3 months after the procedure. The PVC burden and episodes of ventricular arrhythmia were tracked and recorded. All patients underwent routine echocardiographic evaluation immediately after ablation and 3 months later. Long-term success was defined as a reduction in PVC burden of >80% at 3 months after ablation.

### Statistical analysis

Data for continuous variables that were normally distributed are shown as the mean ± standard deviation and those with a skewed distribution as the median (interquartile range). Differences in continuous data were compared between patients with a superior RVOT origin and those with an inferior RVOT origin using the Student's *t-*test or the Kruskal–Wallis rank-sum test as appropriate. Categorical data are shown as the number (percentage) and were compared between the two groups using the chi-squared test. Receiver-operating characteristic (ROC) curve analyses were used to assess the ability of various ECG features to predict an inferior RVOT origin. All statistical analyses were performed using Empowerstats software (version 2.0; Empowerstats, Greenwood Village, CO, USA) and R software (version 3.4.3; R Foundation for Statistical Computing, Vienna, Austria). All analyses were two-sided, and a *P*-value < 0.05 was considered statistically significant.

## Results

### Patient characteristics

The patient demographics, clinical characteristics, and ECG features are shown in Table 1. Seventy-four patients (27 male, 47 female, mean age 47.30 ± 12.86 years) had PVCs with a superior RVOT origin (the superior RVOT group), and 33 (9 male, 24 female, mean age 45.58 ± 15.98 years) had PVCs with an inferior RVOT origin (the inferior RVOT group). There was no significant between-group difference in patient age or sex. Furthermore, all patients had a left ventricular ejection fraction within the normal range (mean, 64.63 ± 7.10% in the inferior RVOT group vs. 65.77 ± 6.94% in the superior RVOT group, *P* = 0.435).

**Table 1:** Patient characteristics and ECG features of PVCs in the superior RVOT group and in the inferior RVOT group.

	**Superior RVOT**	**Inferior RVOT**	**P**
	**(*N =* 74)**	**(*N =* 33)**	
Gender (Female %)	63.51	72.73	0.352
Age	47.30 ± 12.86	45.58 ± 15.98	0.555
PVC burden in 24h Holter (%)	22.87 ± 10.25	24.93 ± 9.05	0.346
LVEF (%)	65.77 ± 6.94	64.63 ± 7.10	0.435
**ECG features**	
Multiform PVCs (%)	6.76	24.24	0.011
The amplitude of lead II (mV)	1.72 ± 0.45	1.21 ± 0.33	<0.001
The amplitude of lead III (mV)	1.62 ± 0.53	0.99 ± 0.46	<0.001
The amplitude of lead aVF (mV)	1.65 ± 0.48	1.09 ± 0.40	<0.001
QRS duration (ms) in sinus rhythm	94.14 ± 10.71	93.55 ± 10.12	0.789
QRS duration (ms) of PVCs	140.90 ± 11.30	162.42 ± 19.69	<0.001
A notch of QRS in Limb leads (%)	30.56	48.48	0.076
A negative delta wave at the onset of the QRS in V1 (%)	1.39	27.27	<0.001
Induced by isoproterenol (%)	16.67	18.18	0.848
Support with long sheath (%)	31.94	51.52	0.055
Successful rate (%)	95.95	87.88	0.199

ECG, electrocardiographic; LVEF, left ventricular ejection fraction; PVC, premature ventricular complexes; RVOT, right ventricular outflow tract; ICE, intracardiac echocardiography.

### ECG features

Patients in the inferior RVOT group were significantly more likely to have multiple morphologies (24.24 vs. 6.76%, *P* = 0.011). The PVC burden was seemingly slightly higher in the inferior RVOT group than in the superior RVOT group (24.93 ± 9.05 vs. 22.87 ± 10.25%, *P* = 0.346). Generally, PVCs with a superior RVOT origin have an inferior axis with LBBB morphology and a late R/S transition in the precordial leads (7). Therefore, a QRS duration <140 ms suggests a PVC with a septal origin, whereas a QRS duration >140 ms favors a free wall origin. However, the QRS wave of PVCs originating from the inferior RVOT does not conform to this rule. Due to the complex anatomic structure of the inferior RVOT, the QRS wave of PVCs originating from this site may be broad and multiform.

Although the morphology of the QRS wave of PVCs was consistent with the characteristics of origin in the RVOT, there were significant differences in QRS amplitude in leads II, III, and aVF between the inferior and superior RVOT groups (see Table 1). The QRS amplitude in the limb leads was lower for PVCs with an inferior RVOT origin than for those with a superior RVOT origin.

Although there was no significant statistical difference in the QRS duration of sinus rhythm between the two groups, PVCs with an inferior RVOT origin were more expansive than those with a superior RVOT origin (QRS duration, 162.42 ± 19.69 ms vs. 140.90 ± 11.30 ms; *P* < 0.001). Furthermore, the QRS wave in the limb leads for PVCs with an inferior RVOT origin was significantly more likely to be with a negative delta wave at the onset of the QRS wave in V1, somewhat like W-P-W (27.27 vs. 1.39%, *P* < 0.001).

The anterior septum was the most common site of origin of PVCs, accounting for 42.42% of PVCs in the inferior RVOT group (14 patients) and 50% of those in the superior RVOT group (37 patients) (see Table 2). In addition to the above ECG features, there was a between-group difference in transition in the precordial leads when the PVCs originated in the anterior septum, in that most of the PVCs arising from the anterior septum transited in V4 and V5 in the inferior RVOT group (78.57%) but transited in V3 and V4 in the superior RVOT group (94.44%) (*P* = 0.036).

**Table 2:** Electrocardiographic characteristics of PVCs originating in the anterior septum in the superior RVOT group and in the inferior RVOT group.

	**Anterior septum of the superior RVOT**	**Anterior septum of the inferior RVOT**	**P**
	**(*N =* 37)**	**(*N =* 14)**	
PVC burden in 24 h Holter (%)	25.10 ± 11.62	24.48 ± 9.54	0.863
**ECG features**	
Multiform PVCs (%)	5.41	7.14	1.000
The amplitude of lead II (mV)	1.62 ± 0.32	1.34 ± 0.37	0.009
The amplitude of lead III (mV)	1.57 ± 0.41	1.23 ± 0.47	0.013
The amplitude of lead aVF (mV)	1.59 ± 0.35	1.31 ± 0.40	0.018
QRS duration (ms) in sinus rhythm	94.35 ± 12.10	93.64 ± 7.43	0.839
QRS duration(ms) of PVCs	139.03 ± 10.54	156.43 ± 13.36	<0.001
A notch of QRS in Limb leads (%)	25.00	35.71	0.449
A negative delta wave at the onset of the QRS in V1 (%)	0	21.43	0.019
Induced by isoproterenol (%)	16.67	21.43	0.697
Support with long sheath (%)	30.56	42.86	0.410
Successful rate (%)	94.59	78.57	0.120

ECG, electrocardiographic; LVEF, left ventricular ejection fraction; PVC, premature ventricular complexes; RVOT, right ventricular outflow tract.

### Radiofrequency ablation

The earliest site of activation of PVCs was 29.3 ± 5.1 ms ahead of the QRS complex in the inferior RVOT group and 27.2 ± 7.0 ms in the superior RVOT group (*P* = 0.572). Therefore, catheter ablation was performed at the site of the earliest activity.

Overall, the ablation success rate was slightly lower for PVCs in the inferior RVOT group than those in the superior RVOT group (87.88 vs. 95.95%, *P* = 0.199). However, there was a difference but it did not reach a statistical significance in the ablation success rate if the PVCs arose from the anterior septum (78.57% in the inferior RVOT group vs. 94.59% in the superior RVOT group, *P* = 0.120) (see Table 2).

It is worth noting that long sheath support was used to maintain the stability of the ablation catheter more often in the inferior RVOT group than in the superior RVOT group (51.52 vs. 31.94%, *P* = 0.055).

### Complex anatomy seen by ICE

**Figure 1:**
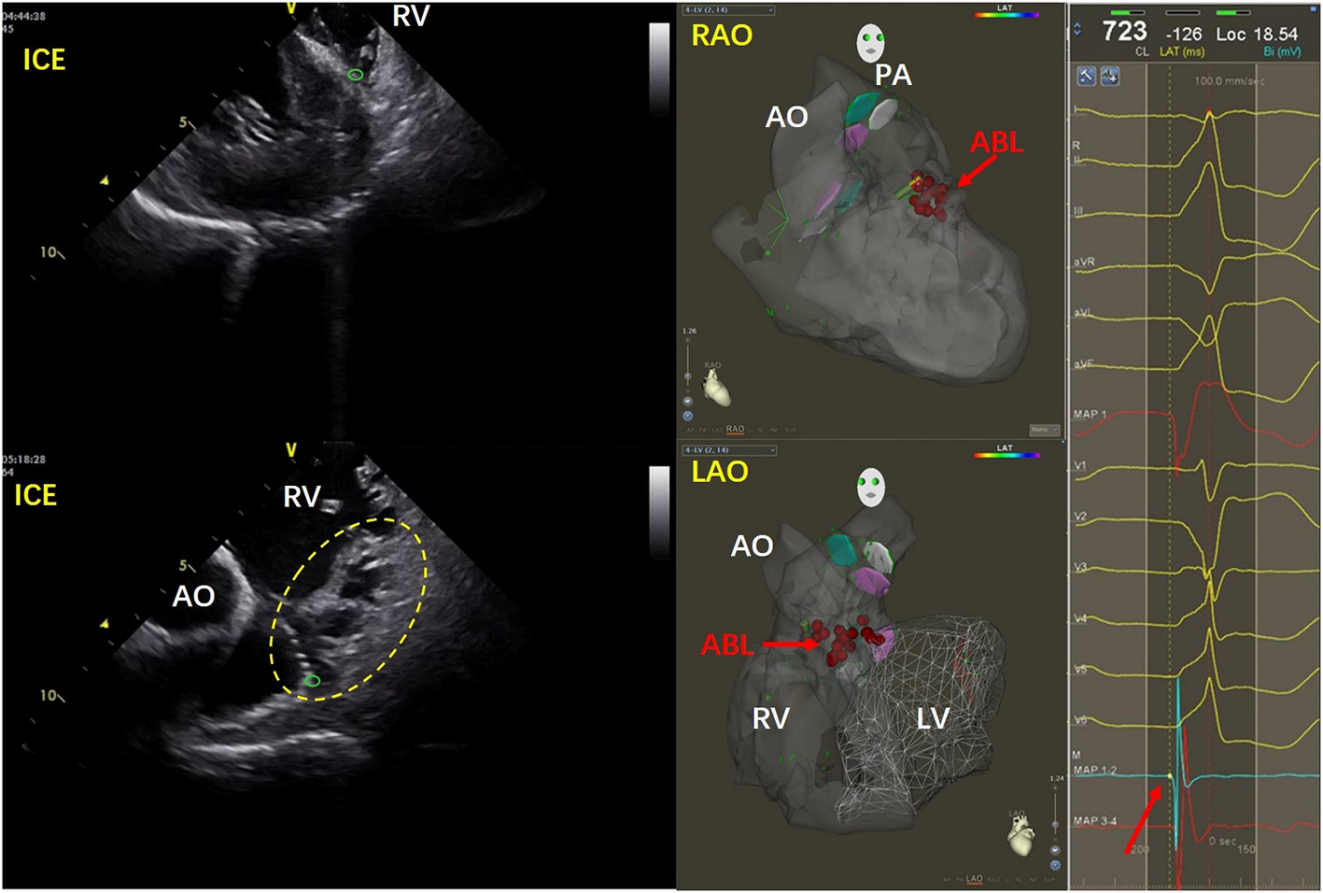
Images for a patient with PVCs that originated from the inferoanterior septum in the inferior RVOT. In the ICE image on the **left**, the tip of the ablation catheter is indicated by the green circle. Abundant trabeculae can be seen in the yellow dotted line area. The tip of the ablation catheter is visible embedded at the intersection of the muscle trabeculae at their junction with the smooth conus of the pulmonary artery. The red arrow in the middle three-dimensional image shows the ablation target. On the **right** side of the figure, the red arrow indicates the early potential at the ablation site. ABL, ablation; AO, aorta; ICE, intracardiac echocardiography; LAO, left anterior oblique; LV, left ventricle; PA, pulmonary artery; RAO, right anterior oblique; RV, right ventricle.

The anatomy of the inferior RVOT is more complex than that of the superior RVOT because it includes the septal papillary muscles, abundant trabeculae, and the moderator band (7). Figure 1 shows a case in which the earliest excitation originated in the lower area of the anterior septum. The ICE image shows the tip of the ablation catheter embedded in the abundant trabeculae where the muscles intersect with the smooth-walled pulmonary infundibulum. In another case, the PVCs originated from the inferoanterior septum of the inferior RVOT. In Supplementary Video 1, the ICE image shows that the tip of the ablation catheter is located at the junction of the interventricular septum and the root of the moderator band. Supplementary Video 2 shows a case of PVCs originating from the middle part of the septum of the inferior RVOT. In the ICE image, the tip of the ablation catheter is inserted into the abundant muscle trabeculae.

### Complications and follow-up

One patient in the inferior group developed pericardial tamponade, whose PVCs originated from the supraventricular crest, the posterior part of the inferior RVOT, where attachment of the catheter is challenging. The patient recovered after immediate pericardial drainage. It is noteworthy that the pericardial tamponade occurred before we started using ICE.

Furthermore, a complete right bundle branch block occurred after ablation in one further patient in the inferior RVOT group and two in the superior RVOT group (3.03 vs. 2.70%, *P* = 0.448).

During a mean follow-up of 8 ± 3 months, there was no statistical difference in the PVC recurrence rate between the inferior RVOT group and the superior RVOT group (9.09 vs. 8.11%, *P* = 0.671). All patients underwent routine echocardiography immediately following catheter ablation and at the 3-month follow-up. There was no change in the left ventricular ejection fraction or any evidence of new or worsening tricuspid regurgitation in any patient.

### Diagnostic value of ECG algorithms for PVCs with an inferior RVOT origin

**Figure 2:**
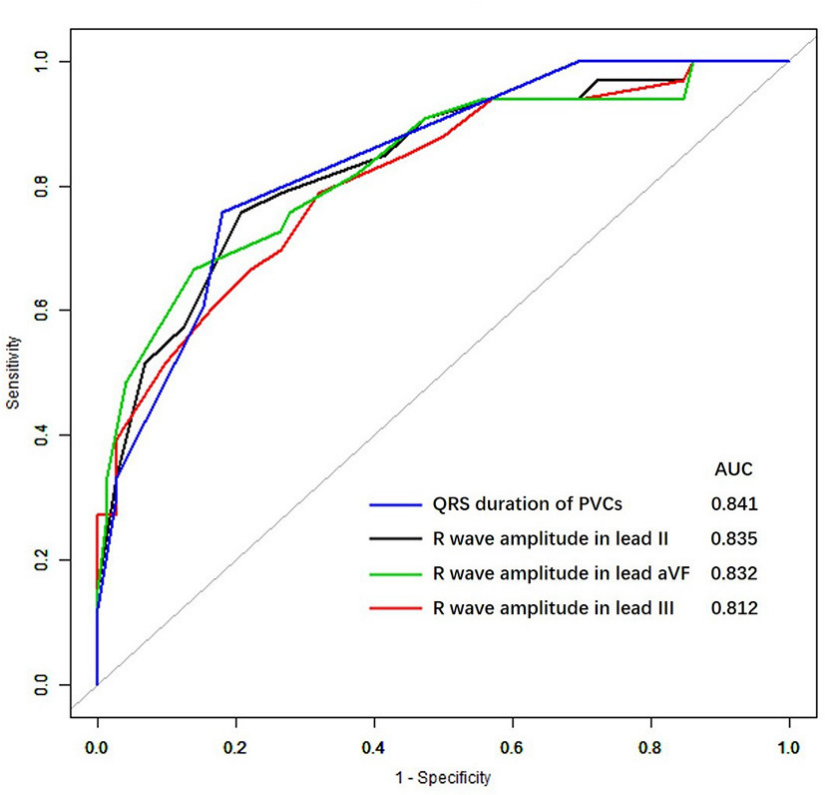
Receiver-operating characteristic curves for premature ventricular complexes in the inferior right ventricular outflow tract. AUC, the area under the receiver-operating characteristic curve.

The areas under the ROC curves (AUCs) for all the previous ECG algorithms used to distinguish PVCs with an inferior RVOT origin from those with a superior RVOT origin were calculated and are shown in Figure 2.

We found that the AUCs for a diagnosis of an inferior RVOT origin were within the range of 0.812–0.841 for various ECG features, including the QRS duration of PVCs, the amplitude of R waves in lead II, III, and aVF; for which the AUC for the QRS duration of PVCs was the highest (0.841).

The ROC curves for the four ECG criteria that differentiated between PVCs of superior RVOT origin and those of inferior RVOT origin (maximum duration of the QRS, the R wave in lead II, the R wave in lead aVF, and the R wave in lead III) are shown in Figure 1. The sensitivities, specificities, positive and negative predictive values, and AUCs for the ability of these criteria to predict the site of origin in the RVOT are shown in Table 3. The R wave amplitude in lead III had the highest sensitivity (79%), and the R wave amplitude in lead aVF had the highest specificity (86%). The positive predictive value for identifying the RVOT region ranged from 53 to 68%, and the negative predictive value ranged from 85 to 88%.

**Table 3:** Best threshold analysis.

**Test**	**Best threshold**	**Specificity**	**Sensitivity**	**Accuracy**	**Positive-PV**	**Negative-PV**
R-wave amplitude in lead II	1.35 mV	0.792	0.758	0.781	0.625	0.877
R-wave amplitude in lead III	1.35 mV	0.681	0.788	0.714	0.531	0.875
R-wave amplitude in lead aVF	1.15 mV	0.861	0.667	0.800	0.688	0.849
QRS duration of PVCs	145 ms	0.819	0.758	0.800	0.658	0.881

Positive-PV, Positive predictive value; Negative-PV, Negative predictive value.

## Discussion

### Main ECG findings

Although Xia et al. have reported ECG indices that can identify whether PVCs originate from the left ventricular outflow tract or the RVOT and Wang et al. have described ECG indices that identify PVCs that originate in the left ventricular outflow tract (9, 10), there is limited information on ECG indices for PVCs originating in the upper and lower RVOT.

PVCs originating from the inferior RVOT have a typical ECG pattern with an LBBB morphology and an inferior axis. Unlike PVCs with a superior RVOT origin that starts around the pulmonary annulus, the QRS of PVCs that originate from the inferior RVOT is upwards in the limb leads but has a lower amplitude and later R/S transition at V4~V5 in the precordial leads.

PVC that originates in the septum of inferior RVOT is characterized by variable morphology, which is consistent with an origin in the RVOT septum but is relatively broad and pleomorphic. PVC originating from the anterior septum in the inferior RVOT often shows a negative delta wave at the onset of the QRS in V1 and polymorphism due to the crisscrossing of the septal papillary muscles, moderator band, and muscle trabeculae at this site. If the QRS wave of V2 is deep and concave, the PVCs may originate from the deep interventricular septum. However, the morphology of PVCs originating from the supraventricular crest of the inferior RVOT can also vary because early Purkinje potentials could be recorded here in some patients (11). A later R/S transition in the precordial leads means that ablation may be more difficult.

**Figure 3:**
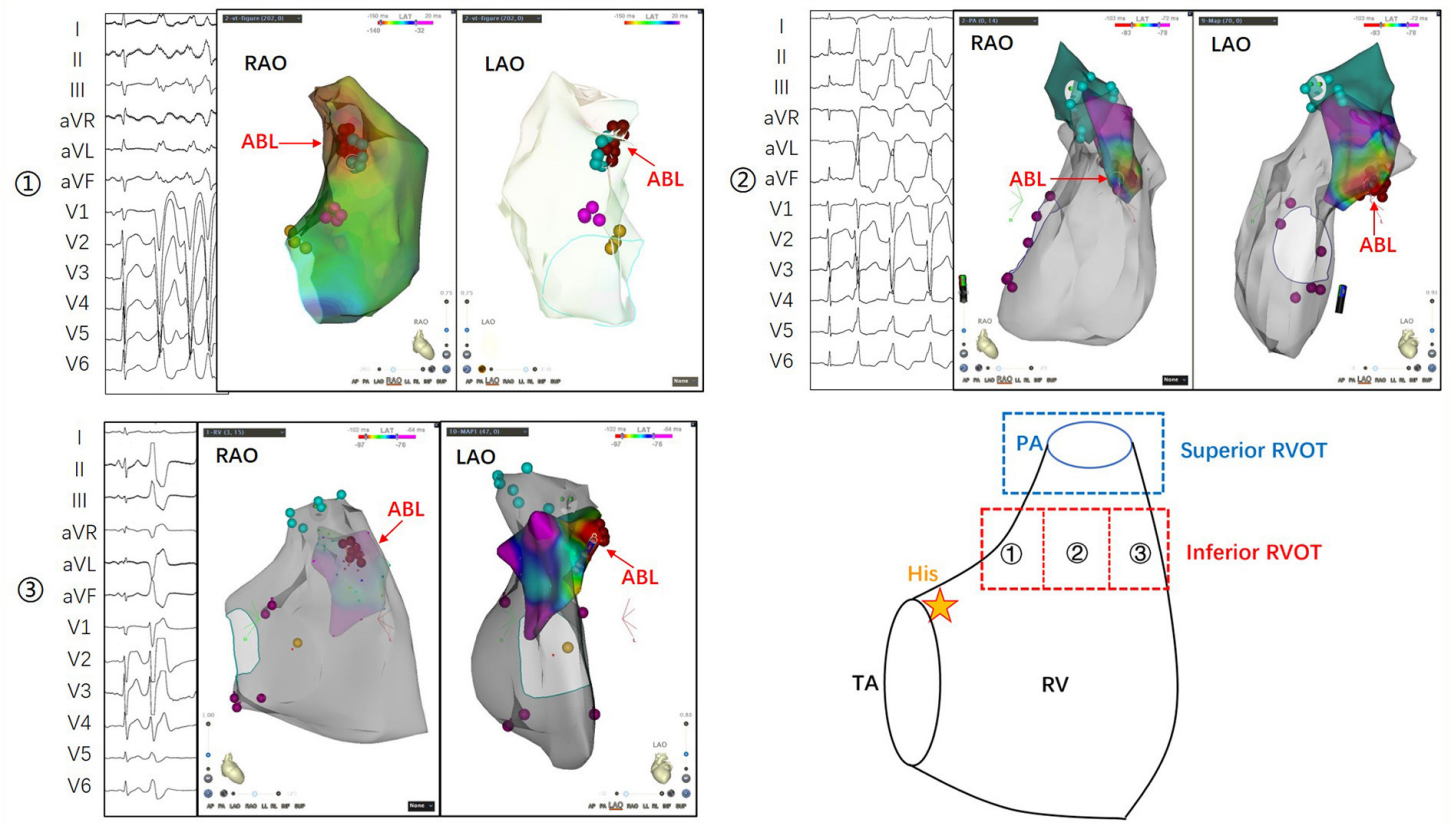
The inferior RVOT is between the supraventricular crest above the tricuspid annulus and the pulmonary annulus. Its lower portion is flush with His bundle, and its upper portion is more than 1 cm lower than the pulmonary annulus. ① The morphological characteristics of PVCs originating from the inferoposterior RVOT include a more comprehensive QRS, an R/S shape in leads II, III, and aVF, a positive main wave in leads I and aVL, and a shift in the R/S transition to between V4 and V5. ② The morphological characteristics of PVCs originating from the inferomedial RVOT include an upright but not tall QRS in leads II, III, and aVF, opposite main waves in leads I and aVL, and a shift of the R/S transition to between V3 and V4. ③ The morphological features of PVCs originating from the inferoanterior RVOT include an upright QRS in leads II, III, and aVF, and a negative main wave in leads I and aVL, indicating the origin of the septum. V1–V3 is QS-shaped, the beginning of the QRS in lead V1 is similar to the preexcitation wave, and the R/S transition is shifted to lead V5. PA, pulmonary artery; RAO, right anterior oblique; RV, right ventricle; RVOT, right ventricular outflow tract; TA, tricuspid annulus.

In summary, the ECG features of PVCs that originate in the inferior RVOT are as follows: (1) although the main waves of QRSs in leads II, III, and aVF are upward, they are relatively low or have S waves; (2) the negative wave is deep in V1–V3 and the R-wave transition is in V4–V5; (3) the QRS wave in V1 may have a negative delta wave at the onset of the QRS (somewhat like the W-P-W waveform); and (4) PVCs may be multiform, or the shape may change during ablation. Typical cases showing the characteristics of PVCs originating from different parts of the inferior RVOT are shown in Figure 3.

We found that the above ECG indices had good predictability for judging the origin of PVCs: (1) the best threshold for the QRS duration in PVCs was 145 ms. and (2) the best thresholds for the amplitude of R waves in leads II, III, and aVF were 1.35 mV, 1.35 mV, and 1.15mV. Thus, the general trend is consistent with the morphological characteristics of RVOT. Furthermore, the QRS wave of the PVCs has a low amplitude and wide duration, which points to an origin in the inferior RVOT.

Based on these ECG characteristics, we can infer the origin of PVCs and select appropriate catheters and additional tools to improve the success rate of ablation.

### Anatomic characteristics of the inferior RVOT

The inferior RVOT is located above the supraventricular crest and below the pulmonary annulus. On the septal aspect, the supraventricular crest inserts between the limbs of the septomarginal trabeculation and the septal band. This muscular strap reinforces the septal surface of the right ventricle, breaking up at the apex to form the moderator band and anterior papillary muscles and giving rise to a further series of septoparietal trabeculations that run to the parietal ventricular wall (7). The anterior portion of the inferior RVOT is connected to the large inflow tract of muscle trabeculae, including the septal papillary muscles and the moderator band. The posterior part of the inferior RVOT contains the conus papillary muscles, supraventricular crest, and the upper edge of the tricuspid annulus. The interventricular septum is adjacent to the left anterior descending coronary artery, and the great cardiac vein runs in the anterior interventricular sulcus.

### Ablation difficulties and techniques

When the ablation target site is located in the right anterior oblique projection, it is flush with or slightly higher than the His bundle. In this situation, there are several main ablation difficulties. First, catheter attachment is difficult. The use of a reversed U-curve ablation catheter is suitable for attachment at a site near the pulmonary annulus but not in the inferior RVOT. Second, there are interspaces between the muscle trabeculae, moderator band, papillary muscles, and the smooth-walled pulmonary conus, where it is difficult to insert a catheter tip accurately without direct vision under ICE. Third, the left anterior descending coronary artery may be damaged by tightening the interventricular septum at its anterior and inferior portions. Fourth, the conduction system in the right bundle branch may be damaged by mechanical compression of the catheter or long sheath.

We recommend the following ablation techniques to overcome some of these difficulties. First, we suggest using a long sheath or steerable sheath to support the ablation catheter. A pressure-sensing ablation catheter can be used to prompt local attachment pressure, which is helpful when judging the attachment. ICE shows the anatomical structure and relationships in real-time, allowing the operator to adjust the moderator band and papillary muscles under direct vision. The tip may become embedded in the muscle trabeculae when the catheter adheres well due to the complex local anatomical structure. Therefore, careful tip pressure adjustment to 10–15 g is essential. If the impedance is still excessive, it is necessary to relax the high impedance limit of the radiofrequency apparatus and increase the heparin saline perfusion speed during catheter ablation. The discharge ablation requires gradual power titration, and PVCs originating from the inferior and anterior portions of the inferior RVOT need to be close to the ventricular septum. If severe pain occurs, the ablation procedure should be stopped because it may damage the left anterior descending coronary artery in the anterior interventricular sulcus or lead to rupture of the ventricular wall.

ICE helps with the judgment of the anatomical location and catheter attachment (12, 13). Furthermore, ICE can be helpful for identifying and confirming contact with the catheter and these endocavitary structures and should always be considered when these structures are potentially involved in the mechanism or substrate of an arrhythmia. Indeed, ICE can enhance the ability to perform successful endocardial mapping and ablation by direct visualization.

## Conclusion

When evaluating a patient with PVCs, an ECG presentation that conforms to the morphological characteristics of RVOT, namely, the QRS wave is relatively broad and polymorphic and the primary waves in limb leads (II, III, and aVF) are upward and have low amplitude, we should consider the inferior RVOT as a likely origin.

## Data availability statement

The original contributions presented in the study are included in the article/Supplementary material, further inquiries can be directed to the corresponding author/s.

## Ethics statement

The studies involving human participants were reviewed and approved by the Ethics Committee of Peking University First Hospital. The patients/participants provided their written informed consent to participate in this study. Written informed consent was obtained from the individual(s) for the publication of any potentially identifiable images or data included in this article.

## Author contributions

All authors listed have made a substantial, direct, and intellectual contribution to the work and approved it for publication.

## Conflict of interest

The authors declare that the research was conducted in the absence of any commercial or financial relationships that could be construed as a potential conflict of interest.

## Publisher's note

All claims expressed in this article are solely those of the authors and do not necessarily represent those of their affiliated organizations, or those of the publisher, the editors and the reviewers. Any product that may be evaluated in this article, or claim that may be made by its manufacturer, is not guaranteed or endorsed by the publisher.

## Supplementary material

Supplementary Video 1 A case in which PVCs originated from the inferoanterior septum of the inferior RVOT. The ICE image on the left shows the tip of the ablation catheter (red arrow) located at the junction of the interventricular septum and the root of the moderator band (pink arrow). The three-dimensional image in the middle shows an RAO projection in which the target can be seen in the inferoanterior portion of the inferior RVOT between the tricuspid annulus and the pulmonary annulus and an LAO projection in which the visual target is located at the root of the moderator band in the interventricular septum. The target map on the right shows the local fragmentation potential of multiple folds. ABL, ablation; ICE, intracardiac echocardiography; LAO, left anterior oblique; LV, left ventricle; MB, moderator bundle; PA, pulmonary artery; RAO, right anterior oblique; RV, right ventricle.

Supplementary Video 2 A case in which PVCs originated from the medial septum of the inferior RVOT. In the ICE image, the tip of the ablation catheter is inserted into the abundant muscle trabeculae. In the radiographic LAO projection, the tip of the ablation catheter is toward the medial septum. In the three-dimensional images, the RAO projection shows the target located in the anterior and upper portions of His bundle and in the middle of the lower RVOT. In the LAO projection, the target is in the ventricular septum. The effective target site shows the early and fragmentation potential. ABL, ablation; His, the bundle of His; ICE, intracardiac echocardiography; LAO, left anterior oblique; LV, left ventricle; MB, moderator band; PA, pulmonary artery; RAO, right anterior oblique; RV, right ventricle.
